# Short reads-based characterization of pathotype diversity and drug resistance among *Escherichia coli* isolated from patients attending regional referral hospitals in Tanzania

**DOI:** 10.1186/s12920-024-01882-y

**Published:** 2024-04-26

**Authors:** Livin E. Kanje, Happiness Kumburu, Davis Kuchaka, Mariana Shayo, Masoud A. Juma, Patrick Kimu, Melkiory Beti, Marco van Zwetselaar, Boaz Wadugu, Blandina T. Mmbaga, Sixbert Isdory Mkumbaye, Tolbert Sonda

**Affiliations:** 1 Department of Microbiology and Immunology, Kilimanjaro Christian Medical University College, Kilimanjaro, Tanzania; 2 Kilimanjaro Clinical Research Institute, Kilimanjaro, Tanzania; 3 Clinical Laboratory, Kilimanjaro Christian Medical Center, Kilimanjaro, Tanzania; 4 State University of Zanzibar, Zanzibar, Tanzania

**Keywords:** Pathotypes, *E. Coli*, Diversity, Drug resistance

## Abstract

**Background:**

*Escherichia coli* is known to cause about 2 million deaths annually of which diarrhea infection is leading and typically occurs in children under 5 years old. Although Africa is the most affected region there is little information on their pathotypes diversity and their antimicrobial resistance.

**Objective:**

To determine the pathotype diversity and antimicrobial resistance among *E. coli* from patients attending regional referral hospitals in Tanzania.

**Materials and methods:**

A retrospective cross-section laboratory-based study where a total of 138 archived *E. coli* isolates collected from 2020 to 2021 from selected regional referral hospitals in Tanzania were sequenced using the Illumina Nextseq550 sequencer platform. Analysis of the sequences was done in the CGE tool for the identification of resistance genes and virulence genes. SPSS version 20 was used to summarize data using frequency and proportion.

**Results:**

Among all 138 sequenced *E. coli* isolates, the most prevalent observed pathotype virulence genes were of extraintestinal *E. coli* UPEC *fyuA* gene 82.6% (114/138) and NMEC *irp* gene 81.9% (113/138). Most of the *E. coli* pathotypes observed exist as a hybrid due to gene overlapping, the most prevalent pathotypes observed were NMEC/UPEC hybrid 29.7% (41/138), NMEC/UPEC/EAEC hybrid 26.1% (36/138), NMEC/UPEC/DAEC hybrid 18.1% (25/138) and EAEC 15.2% (21/138). Overall most *E. coli* carried resistance gene to ampicillin 90.6% (125/138), trimethoprim 85.5% (118/138), tetracycline 79.9% (110/138), ciprofloxacin 76.1% (105/138) and 72.5% (100/138) Nalidixic acid. Hybrid pathotypes were more resistant than non-hybrid pathotypes.

**Conclusion:**

Whole genome sequencing reveals the presence of hybrid pathotypes with increased drug resistance among *E. coli* isolated from regional referral hospitals in Tanzania.

**Supplementary Information:**

The online version contains supplementary material available at 10.1186/s12920-024-01882-y.

## Introduction

*Escherichia coli* is a gram-negative rod-shaped bacterium from the family *Enterobacteriaceae* known to cause a variety of diseases ranging from diarrheagenic to extraintestinal infections due to their pathogenic effects and productions of various toxins and other virulence factor [1]. It is known to cause about 2 million deaths annually as a result of diarrhea infection typically occurring in children under 5 years old and mostly affecting tropical and sub-tropical regions [2]. In Dar es Salaam Tanzania, a previous study reported that about 51.1% of UTI cases are caused by *E. coli* [3]. Similarly, in the northern part of Tanzania *E. coli* account for 46.2% of all UTI cases among children [4]. Another study conducted in HIV-infected people in the northern part of Tanzania reported the prevalence of bacteriuria in HIV patients 12.3% whereby 16.2% of the causative agent is *E. coli* [5]. It has also been demonstrated that *E. coli* causes about 11.7% of the human bacterial infection [6].

World Health Organization reorganizes antimicrobial resistance as among the top 10 global public health threats facing humanity today. Resistance bacterial infections are associated with 4.95 million deaths per year [7]. Antimicrobial resistance increases healthcare costs due to more expensive therapy, prolonged hospitalization, and high morbidity rates [8]. It is estimated that by 2050, drug resistance will cause more deaths than all cancers combined [9].

Ability of the pathogenic *E. coli* to cause a variety of diseases from intestinal to extra-intestinal infection is a result of a unique acquired virulence factor that differentiates *E. coli* into pathotypes based on the specific disease and the site of infections [10–12]. Intestinal *E. coli* pathotypes include; Enterotoxigenic *E. coli* (ETEC), Enteropathogenic *E. coli* (EPEC), Shiga toxin-producing *E. coli* (STEC), Enteroinvasive *E. coli* (EIEC), Enteroaggregative *E. coli* (EAEC) and diffusely adherent *E. coli* (DAEC) [11] while Extraintestinal *E. coli* pathotypes are; Neonatal meningitis *E. coli* (NMEC), Uropathogenic *E. coli* (UPEC) and Septicemic *E. coli* [1, 13].

Horizontal gene transfer of the resistance and virulence genes among different *E. coli* increases antimicrobial resistance and creates diversity. In addition, hybrid pathotypes may result from gene overlapping processes [14–16]. Hybrid pathotypes cause more severe disease and complications. Evidently, a single *E. coli* has been reported to cause both diarrhea and hemolytic uremic syndrome [17]. Another report suggested that 93.5% (101/108) of the *E. coli* isolates that were originally known as intestinal *E. coli* carry genes also for extraintestinal *E. coli* [18].

Despite of the significance of next-generation sequencing in analyzing pathogenic bacteria, their utilization is rare in low- and middle-income countries like Tanzania, leading to a scarcity of information regarding diversity among *E. coli* pathotypes. Whole genome sequencing (WGS) of the particular pathogen provides precise information on the species identity, resistance gene, plasmid, virulence gene, multi-locus sequence typing (MLST), or serotyping compared to other methods [19, 20]. Understanding diversity among the pathotypes is important for determining the genetic relatedness and variability among the strains while assessing drug resistance patterns is essential for guiding appropriate treatment strategies. This study aims to determine the level of antimicrobial resistance and pathotype diversity among *E. coli* isolated from regional referral hospitals in Tanzania.

## Materials and methods

### Study design, study participants, and study sites

This was a retrospective cross-section laboratory-based study using achieved *E. coli* culture sample isolated from urine, wound, pus, blood, sputum, and stool collected from January 2020 to December 2021 at Regional Referral Hospital of Tanzania. This study was nested from SeqAfrica project with the main objective of developing, expanding, and supporting whole genome sequencing and bioinformatics capacity for antimicrobial resistance surveillance across Africa. The project is functioning in four countries across Africa; Ghana, Nigeria, South Africa, and Tanzania. In Tanzania six study areas were selected; Tabora, Dodoma, Songea, Kigoma, Morogoro, and Zanzibar.

### DNA extraction and whole genomic sequencing

DNA from *E. coli* strains was extracted using Quick-DNA™ Fungal/Bacteria Miniprep Kit as per manufacturer instructions. The purity and the quantity of the extracted DNA were checked by using a Qubit® version 4.0 fluorometer. Library preparation was performed based on the NEBNext® Ultra™ II FS DNA Library Prep Kit manual 2020. Briefly, library preparation involves fragmentation, adaptor ligation, size selection, and indexing or barcoding of each extracted DNA from different *E. coli*. The prepared library was normalized and combined with Phix control before loading in the Illumina Nextseq550 sequencer platform for sequencing.

### Bioinformatics analysis

Quality control of the sequenced raw data was performed using FastQC 0.12.0 [21]. De novo assembly was performed using SPAdes 3.15.5 [22] and the final output files were in Fasta format. Bacterial Analysis Pipeline (BAP 3.3.2) which is based on the services available at the Center for Genomic Epidemiology (CGE) (https://www.genomicepidemiology.org/services/) was used. Species identification was determined using Kmerfinder 3.2 [23–25], the Resistance gene was determined using Resfinder 4.1 [26–28] and the virulence gene was determined using VirulenceFinder 2.0 [26, 29, 30]. The virulence genes that were used to define *E. coli* pathotypes are presented in supplementary Table 1. The assembled *E. coli* genome from this study has been submitted to the European Nucleotide Archive with project accession number PRJEB71714. SPSS version 20 was used to summarize data using frequency and proportion.

## Results

### Distribution of *E. Coli* across the hospitals

A total of 138 non-duplicate *E. coli* isolates were retrieved of which 60 (43.5%) were from urine, 45 (32.6%) from pus wound swabs, 15 (10.9%) from high vaginal swabs, and 13 (9.4%) from blood. 48 (34.8%) of the *E. coli* isolates were collected from Dodoma regional referral hospitals, 36 (26.1%) from Morogoro regional referral hospitals, and only 5 (3.6%) from Kigoma regional referral hospitals. The majority 136 (98.6%) of the isolates were cultured in 2021 and half of the participants (50.0%) were aged 18–34 years old (Table 1).

**Table 1:** Distribution of *E. coli* across the hospitals

Variable	Frequency (n)	(n/N) %
Origin of sample		
Stool	3	2.2
Blood	13	9.4
Peritoneal fluid	1	0.7
High vaginal swab	15	10.9
Urine	60	43.5
Pus wound swab	45	32.6
Sputum	1	0.7
Age		
< 18	7	5.1
18–34	69	50.0
> 34	62	44.9
Mean (± SD)	36.06 (± 15.06)	
Cultured date		
2020	2	1.4
2021	136	98.6
Health Facility		
Dodoma Regional Referral Hospital	48	34.8
Morogoro Regional Referral Hospital	36	26.1
Zanzibar Regional Referral Hospital	17	12.3
Tabora Regional Referral Hospital	15	10.9
Songea Regional Referral Hospital	17	12.3
Kigoma Regional Referral Hospital	5	3.6
**Total**	**138**	**100**

### Antimicrobial resistance genes and predicted phenotypic resistance profiles

Among all 138 sequenced *E. coli*, 125 (90.6%) were harboring at least one of *blaOXA-1, blaTEM-1B*, and *blaCTX-M-15* gene which can confer resistance to resistant to ampicillin, 118 (85.5%) were harboring *dfrA1, dfrA5, dfrA7, dfrA8, dfrA12, dfrA14, and dfrA1 gene* which can confer resistance to resistant to trimethoprim, 110 (79.7%) were harboring at least one of *tet(A), tet(B) and tet(D)* gene which can confer resistance to tetracycline, 105 (76.1%) were harboring at least one of *aac(6’)-Ib-cr* gene, *qnrS1* gene, *gyrA* mutation,, *parC* mutation and *parE* mutation which can confer resistance to ciprofloxacin and 100 (71.9%) were harboring at least one of *qnrS1* gene, *gyrA* mutation, *parC* mutation and *parE* mutation which can confer resistance to Nalidixic acid. (Table 2).

**Table 2:** Antimicrobial resistance genes and predicted phenotypic resistance profiles (*N* = 138)

Antibiotic class	*AMR gene	Resistance due to Mutation	Antibiotics	Resistant-Isolate, n (%)
Aminoglycoside	*aac(3)-IId*	-	Gentamicin	52 (37.7)
Tetracyclines	*tet(A), tet(B), tet(D)*	-	Tetracycline	110 (79.7)
Folate synthesis-inhibitor	*dfrA1, dfrA5, dfrA7, dfrA8, dfrA12, dfrA14* and *dfrA17*	-	trimethoprim	118 (85.5)
Beta-Lactam	*BlaNDM-5, blaOXA-1, blaTEM-1B, blaCTX-M-15* and *blaCTX-M-27*	-	Meropenem Amoxicillin/clavunated Ampicillin Ceftriaxone Ceftazidime	2 (1.4) 41 (29.7) 125 (90.6) 68 (49.3) 68 (49.3)
Quinolone	*qnrS1*	*gyrA* mutation, *parC* mutation and *parE* mutation	Ciprofloxacin Nalidixic acid	105 (76.1) 100 (72.5)
Amphenicol	*cmlA1, cat, catA1, catA2, catB3* and *floR*	-	Chloramphenicol	18 (13.0)

*AMR gene- Antimicrobial resistance gene

### *E. Coli* pathotypes virulence genes

Among all sequenced *E. coli* isolate, 114 (82.6%) were harboring the *fyuA* virulence gene for UPEC pathotype, 113 (81.9%) were harboring *irp* virulence gene for NMEC pathotype, 95 (68.8%) were harboring *chuA* virulence gene for UPEC, 80 (58.0%) were harboring *yfcV* virulence gene for UPEC, and 71 (51.4) were harboring *hlyE* virulence gene for EAEC (Table 3).

**Table 3:** *E. coli* pathotypes virulence genes (*N* = 138)

Pathotypes	Virulence gene	Prevalence, n (%)
Diarrheagenic *E. coli* (DEC)		
EAEC	*aggR*	3 (2.2)
	*aatA*	3 (2.2)
	*agg3A*	3 (2.2)
	*hlyE*	71 (51.4)
DAEC	*afaA/E*	37 (26.8)
EIEC	*ipaH*	1 (0.7)
EPEC	*espA*	1 (0.7)
	*eae*	1 (0.7)
EHEC	*Stx1, stx2*	0 (0.0)
Extraintestinal *E. coli* (ExPEC)		
UPEC	*fyuA*	114 (82.6)
	*chuA*	95 (68.8)
	*yfcV*	80 (58.0)
	*vat*	16 (11.6)
	*papC*	46 (33.3)
	*sfa*	5 (3.6)
	*cnf*	24 (17.4)
NMEC	*irp*	113 (81.9)
	*neuC*	16 (11.6)

### Predicted *E. Coli* pathotypes using virulence gene

Most of the *E. coli* pathotypes observed from sequenced isolates exist as a hybrid due to gene overlapping, the most prevalent pathotypes observed were 41 (29.7%) NMEC/UPEC hybrid, 36 (26.1%) NMEC/UPEC/EAEC hybrid, 25 (18.1%) NMEC/UPEC/DAEC hybrid, and 21 (15.2%) EAEC. The least observed pathotypes were 1 (0.7%) UPEC, 1 (0.7%) EPEC/EAEC hybrid, 1 (0.7%) UPEC/EAEC/DAEC hybrid, and 1 (0.7%) NMEC/UPEC/EAEC/EIEC hybrid (Table 4).

**Table 4:** Predicted *E. coli* pathotypes using virulence genes (*N* = 138)

Pathotype	Frequency (n)	(n/N) %
EAEC	21	15.2
UPEC	1	0.7
EPEC/EAEC Hybrid	1	0.7
NMEC/UPEC Hybrid	41	29.7
NMEC/UPEC/DAEC Hybrid	25	18.1
NMEC/UPEC/EAEC Hybrid	36	26.1
NMEC/UPEC/EAEC/DAEC Hybrid	11	8.0
NMEC/UPEC/EAEC/EIEC Hybrid	1	0.7
UPEC/EAEC/DAEC Hybrid	1	0.7
**Total**	**138**	**100**

### Association between *E. Coli* pathotypes and their antimicrobial resistance profiles

Among all pathotypes, high resistance was observed for the antibiotic ampicillin and trimethoprim in which the hybrid pathotypes were more resistant than non-hybrid pathotypes. All pathotypes were sensitive to the antibiotic meropenem with a slight resistance of 2.4% and 2.8% among the NMEC/UPEC hybrid and NMEC/UPEC/EAEC hybrid respectively (Table 5).

**Table 5:** *E. coli* pathotypes with drug resistance

PATHOTYPES (n)	CRO	TRM	GEN	AMP	CIP	AMC	TET	CHL	CAZ	MEM	NA
EAEC (*n*= 21)	4 (19.0%)	13 (61.9%)	3 (14.3%)	12 (57.1%)	10 (47.6%)	4 (19.0%)	16 (76.2%)	3 (14.3%)	4 (19.0%)	0 (0.0%)	6 (28.6%)
EPEC/EAEC (*n*= 1)	1 (100%)	1 (100%)	0 (0.0%)	1 (100%)	1 (100%)	0 (0.0%)	1 (100%)	1 (100%)	1 (100%)	0 (0.0%)	1 (100%)
NMEC/UPEC (*n*= 41)	16 (39.0%)	37 (90.2%)	21 (51.2%)	39 (95.1%)	37 (90.2%)	6 (14.6%)	36 (87.8%)	1 (2.4%)	16 (39.0%)	1 (2.4%)	37 (90.2%)
NMEC/UPEC/DAEC (*n*= 25)	18 (72.0%)	23 (92.0%)	16 (64.0%)	25 (100%)	20 (80.0%)	14 (56.0%)	19 (76.0%)	1 (4.0%)	18 (72.0%)	0 (0.0%)	20 (80.0%)
NMEC/UPEC/EAEC (*n*= 36)	17 (47.2%)	30 (83.3%)	6 (16.7%)	34 (94.4%)	27 (75.0%)	8 (22.2%)	30 (83.3%)	10 (27.8%)	17 (47.2%)	1 (2.8%)	26 (72.2%)
NMEC/UPEC/EAEC/DAEC (*n*= 11)	9 (81.8%)	11 (100%)	6 (54.5%)	11 (100%)	9 (81.8%)	9 (81.8%)	6 (54.5%)	0 (0.0%)	9 (81.8%)	0 (0.0%)	9 (81.8%)
NMEC/UPEC/EAEC/EIEC (*n*= 1)	1 (100%)	1 (100%)	0 (0.0%)	1 (100%)	1 (100%)	0 (0.0%)	1 (100%)	0 (0.0%)	1 (100%)	0 (0.0%)	1 (100%)
UPEC (*n*=1)	1 (100%)	1 (100%)	0 (0.0%)	1 (100%)	0 (0.0%)	0 (0.0%)	0 (0.0%)	1 (100%)	1 (100%)	0 (0.0%)	0 (0.0%)
UPEC/EAEC/DAEC (*n*= 1)	1 (100%)	1 (100%)	0 (0.0%)	1 (100%)	0 (0.0%)	0 (0.0%)	1 (100%)	1 (100%)	1 (100%)	0 (0.0%)	0 (0.0%)
**Total**	68 (48.9%)	119 (85.6%)	52 (37.4%)	126 (90.6)	105 (75.5%)	41 (29.5%)	110(79.1%)	18 (12.9%)	68 (48.9%)	2 (1.4%)	100 (71.9%)

CHL- Chloramphenicol, MEM- Meropenem, CIP- Ciprofloxacin, AMC- Amoxicillin-clavunated, AMP- Ampicillin, CAZ- Ceftazidime, TMP-Trimethoprim, CRO- Ceftriaxone, GEN- Gentamicin, NAL- Nalidixic acid

## Discussion

The present study aimed to determine the diversity among the *E. coli* pathotypes and their associated drug resistance in regional referral hospitals in Tanzania by the use of whole genome sequencing technology. High diversity in the *E. coli* pathotypes was observed in which extra-intestinal *E. coli* virulence genes for UPEC and NMEC pathotypes were most prevalent while the least virulence genes were of diarrheagenic *E. coli* pathotypes (Table 3.). These specific virulence genes together with other factors such as tissue tropisms, pathogenesis, and clinical features as explained by [1, 31, 32] are what divide *E. coli* into either intestinal or extraintestinal pathotypes. But also, the highly prevalent UPEC reported in this study might be due to more urine samples that were used in this study. Horizontal gene transfer of the specific pathotyped virulence factor can cause diversity and formation of hybrid pathotypes [15, 16].

As a result of virulence gene overlapping reported in this study, seven different types of hybrid pathotypes were observed (Table 4). The only pathotypes that were not hybrid were EAEC and UPEC. The combination of virulent factors to form hybrid or hetero-hybrid pathotypes results in more severity of the disease by creating more complications [33]. A similar case was also reported by [17] where a single *E. coli* strain causes both diarrhea and hemolytic uremic syndrome which creates more complications and questions on the best therapy to use. Another study from Norway reported that 93.5% (101/108) of the *E. coli* isolates that were originally known as intestinal *E. coli* carry genes also for extra-intestinal *E. coli* [18].

Following analysis for resistance and prediction of phenotypic resistance among all pathotypes, hybrid pathotypes were more resistant than non-hybrid in which high resistance was observed for the antibiotic ampicillin, trimethoprim ciprofloxacin, tetracycline, and nalidixic acid. All pathotypes were sensitive to the antibiotic meropenem with a slight resistance of 2.4% and 2.8% among the NMEC/UPEC hybrid and NMEC/UPEC/EAEC hybrid respectively (Table 5). Overall, most *E. coli* isolates carried resistance genes for ampicillin (90.6%) (*blaOXA-1, blaTEM-1B* and *blaCTX-M-15*), trimethoprim (85.5%) (*dfrA1, dfrA5, dfrA7, dfrA8, dfrA12, dfrA14 and dfrA17*), tetracycline (79.7%) (*tet(A), tet(B) and tet(D)*) and ciprofloxacin (76.1%) (*aac(6’)-Ib-cr, gyrA* mutation, *qnrS1, parC* mutation and *parE* mutation) and (71.9%) Nalidixic acid (*qnrS1, gyrA* mutation, *parC* mutation and *parE* mutation). A longitudinal assessment of antibiotic resistance in fecal *E. coli* in Tanzanian children also reports high resistance to ampicillin but low resistance to ciprofloxacin [34]. Ciprofloxacin was reported as among the most used antibiotics by 9.27 (6.0%) Daily Doses per 1000 inhabitants per day from 2010 to 2016 [35] which could be the factor for the observed increase in the resistance.

Resistance gene *mcr-1* and *blaCTX-M-15*-like were also reported in Zanzibar among the sequenced *E. coli* with a prevalence of 55% and 51.0% respectively. These resistance genes code for colistin and extended-spectrum cephalosporines resistance genes respectively [36, 37]. Another study conducted in Tanzania also reported that 63% (77/123) of gram-negative bacteria carry resistance gene *dfrA* of which *dfrA1* was frequently in *E. coli* isolates [38].

Despite the observed high resistance gene among the sequenced *E. coli*, Meropenem, Amoxicillin-clavunated and Gentamicin antibiotics were less occupied with the resistance gene with the resistance prevalence of 1.4%, 29.7%, and 37.7% respectively. This brings hope for an alternative drug of choice for treatment of the *E. coli* infection despite the reduced treatment options for *E. coli* infections.

Furthermore, the resistance gene observed in the present study was observed to cause resistance in more than one antibiotic of either one or different class, this was also reported in other studies such that; the *aac(6’)-Ib-c* gene was reported to cause antibiotic resistance to aminoglycoside antibiotic amikacin and quinolone antibiotic ciprofloxacin [39, 40]; *gyrA* gene mutation can also confer resistance to nalidixic acid and ciprofloxacin antibiotics of which they both belong to quinolone antibiotic group [41, 42]. The clinical implications for the observed results are that; treatment options and management for the *E. coli* infection should be reviewed mostly in low- and middle-income countries. Additionally, observed findings suggest the proper use of antibiotics to reduce the development of drug resistance and disease severity.

## Conclusion

There is a reduced treatment option for *E. coli* infections due to increased drug resistance, the only drug with less observed resistance in our setting was meropenem 1.4% a third generation β-lactam antibiotic of the carbapenem class. There is an increase in *E. coli* pathotype diversity, severity, and complications of the disease due to the large number of observed pathotype hybrid.

### Electronic supplementary material

Below is the link to the electronic supplementary material.

Supplementary Material 1

## Data Availability

Assembled E. coli genome from this study have been submitted to European Nucleotide Archive with project accession number PRJEB71714.
