# Supplementary material for: Short reads-based characterization of pathotype diversity and drug resistance among Escherichia coli isolated from patients attending regional referral hospitals in Tanzania
Source: BMC Med Genomics. 2024 Apr 26;17:110. doi: 10.1186/s12920-024-01882-y (PMC11055328; doi:10.1186/s12920-024-01882-y)
Supplement: Supplementary file 1 — Supplementary Material 1 [file 12920_2024_1882_MOESM1_ESM.docx]

**Supplementary Table 1.** Virulence gene defining *E. coli* pathotypes

| Pathotypes | Virulence gene | References |
| --- | --- | --- |
| Diarrheagenic *E. coli* (DEC) |  |  |
| EAEC | *aggR*  *aatA*  *agg3A*  *hlyE* | [45]  [18]  [35] |
| DAEC | *afaA/E* | [35] |
| EIEC | *ipaH* | [45] |
| EPEC | *espA*  *eae* | [46] |
| EHEC | *Stx1 and stx2* | [47] |
| Extraintestinal *E. coli* (ExPEC) |  |  |
| UPEC | *chuA*  *yfcV*  *vat*  *papC*  *sfa*  *cnf* | [35]  [48] |
| NMEC | *irp*  *neuC* | [49]  [50] |
